# Secondary endpoints analysis in patients with estrogen receptor-positive metastatic breast cancer treated with everolimus and exemestane enrolled in Oral Care-BC

**DOI:** 10.1186/s12885-020-07746-9

**Published:** 2021-01-07

**Authors:** Katsuhiko Nakatsukasa, Naoki Niikura, Kosuke Kashiwabara, Takeshi Amemiya, Ken-ichi Watanabe, Hironobu Hata, Yuichiro Kikawa, Naoki Taniike, Takashi Yamanaka, Sachiyo Mitsunaga, Kazuhiko Nakagami, Moriyasu Adachi, Naoto Kondo, Yasuyuki Shibuya, Naoki Hayashi, Mariko Naito, Toshinari Yamashita, Masahiro Umeda, Hirofumi Mukai, Yoshihide Ota

**Affiliations:** 1 Department of Endocrine and Breast Surgery, Kyoto Prefectural University of Medicine, 465 Kajii-cho, Kawaramachi-hirokoji, Kamigyo-ku, Kyoto City, 602-8566 Japan; 2 Department of Breast and Endocrine Surgery, Tokai University School of Medicine, Tokyo, Japan; 3 Department of Biostatistics, School of Public Health, The University of Tokyo, Tokyo, Japan; 4 Department of Dentistry and Oral and Maxillofacial Surgery, Kyoto Prefectural University of Medicine, Kyoto, Japan; 5 Department of Breast Surgery, Hokkaido Cancer Center, Sapporo, Japan; 6 Department of Dentistry, Hokkaido Cancer Center, Sapporo, Japan; 7 Department of Breast Surgery, Kobe City Medical Center General Hospital, Kobe, Japan; 8 Department of Dentistry and Oral and Maxillofacial Surgery, Kobe City Medical Center General Hospital, Kobe, Japan; 9 Department of Breast and Endocrine Surgery, Kanagawa Cancer Center, Yokohama, Japan; 10 Department of Dentistry and Oral and Maxillofacial Surgery, Kanagawa Cancer Center, Yokohama, Japan; 11 Department of Breast and Endocrine Surgery, Shizuoka General Hospital, Shizuoka, Japan; 12 Department of Oral and Maxillofacial Surgery, Shizuoka General Hospital, Shizuoka, Japan; 13 Department of Breast and Endocrine Surgery, Nagoya City University Hospital, Nagoya, Japan; 14 Department of Dentistry and Oral and Maxillofacial Surgery, Nagoya City University Hospital, Nagoya, Japan; 15 Department of Breast Surgical Oncology, St. Luke’s International Hospital, Tokyo, Japan; 16 Department of Oral Epidemiology, Graduate School of Biomedical and Health Sciences Hiroshima University, Hiroshima, Japan; 17 Department of Clinical Oral Oncology, Nagasaki University Graduate School of Biomedical Sciences, Nagasaki, Japan; 18 Department of Breast and Medical Oncology, National Cancer Center Hospital East, Kashiwa, Japan; 19 Department of Dentistry and Oral and Maxillofacial Surgery, Tokai University School of Medicine, Tokyo, Japan

**Keywords:** Breast cancer, Everolimus, Progression-free survival, Oral care, Oral care-BC

## Abstract

**Background:**

The Oral Care BC-trial reported that professional oral care (POC) reduces the incidence and severity of oral mucositis in patients receiving everolimus (EVE) and exemestane (EXE). However, the effect of POC on clinical response among patients receiving EVE and EXE was not established. We compared outcomes for estrogen receptor-positive metastatic breast cancer patients who received POC to those who had not, and evaluated clinical prognostic factors. All patients simultaneously received EVE and EXE.

**Methods:**

Between May 2015 and Dec 2017, 174 eligible patients were enrolled in the Oral Care-BC trial. The primary endpoint was the comparative incidence of grade 1 or worse oral mucositis, as evaluated for both the groups over 8 weeks by an oncologist. The secondary endpoints were progression-free survival (PFS) and overall survival (OS). Data were collected after a follow-up period of 13.9 months.

**Results:**

There were no significant differences in PFS between the POC and Control Groups (*P* = 0.801). A BMI <  25 mg/m^2^ and non-visceral metastasis were associated with longer PFS (*P* = 0.018 and *P* = 0.003, respectively) and the use of bone modifying agents (BMA) was associated with shorter PFS (*P* = 0.028). The PFS and OS between the POC and control groups were not significantly different in the Oral-Care BC trial.

**Conclusions:**

POC did not influence the prognosis of estrogen receptor-positive metastatic breast cancer patients. Patients with non-visceral metastasis, a BMI <  25 mg/m^2^, and who did not receive BMA while receiving EVE and EXE may have better prognoses.

**Trial registration:**

The study protocol was registered online at the University Hospital Medical Information Network (UMIN), Japan (protocol ID 000016109), on January 5, 2015 and at ClinicalTrials.gov (NCT02376985).

## Background

Everolimus (EVE), an oral mammalian rapamycin (mTOR) inhibitor, exerts antitumor effects against various cancers including breast cancer and renal cell carcinoma [1, 2]. Since the BOLERO-2 study, EVE and exemestane (EXE) have been approved by the US Food and Drug Administration for use in patients with estrogen receptor (ER)-positive metastatic breast cancer [3, 4] and this combination has been investigated extensively [5–8].

Oral Care-BC was a phase 3 multicenter randomized clinical trial that assessed the effectiveness of professional oral care (POC) in preventing oral mucositis in patients treated with EVE and EXE for hormone-receptor-positive HER2-negative metastatic breast cancer. We previously reported that POC reduces the incidence and severity of oral mucositis in patients receiving EVE and EXE and that POC significantly reduces the incidence of grade 1 and 2 oral mucositis within 8 weeks [9].

However, the population with better clinical response among patients receiving EVE and EXE was not established. Biomarker analyses have been conducted with the goal of identifying subsets of patients that may benefit from EVE treatment [10–13]. Therapeutic drugs such EVE can cause oral mucositis, thus inducing pain and other associated symptoms. Difficulties in oral ingestion and poor oral hygiene increases bacteria inside the oral cavity, which is associated with systemic risks due to increased risk of aspiration pneumonitis. Oral mucositis interferes with treatment and prolongs hospitalization [14, 15]. Furthermore, oral mucositis can result in the discontinuation of treatment. Therefore, we thought that decreasing oral mucositis by POC could result in better survival parameters, besides alleviating mucositis. We hypothesized that POC might have a good prognostic effect in patients treated with EVE. This is the first randomized trial to compare POC with a control to evaluate its effectiveness in reducing oral mucositis.

## Methods

### Consort

This study design adhered to CONSORT guidelines. The Consort Flow Chart has been provided in the article on primary endpoints [9].

### Patients

#### Study design

Using a dynamic allocation method that minimizes the effects of the allocation adjustment factors discussed below, the Comprehensive Support Project for Oncology Research (CSPOR) Data Center randomly assigned treatment protocols to subjects categorized into the POC and Control (C) groups in a ratio of approximately 1:1. The allocation algorithm was used by the researcher responsible for biostatistical analysis who considered the following factors: age at enrollment (< 65 years / ≥ 65 years); use of bone-modifying agents (yes / no); receiving chemotherapy within the last 3 months (yes / no); and institution name.

Patients were randomly assigned in a 1:1 ratio (stratified according to center, use of BMA, age, and history of receiving chemotherapy within 3 months) [9]. Eligible patients were enrolled at 31 investigation sites from academic and community settings in Japan on the basis of the following key inclusion criteria: postmenopausal women having histologically or cytologically confirmed metastatic hormone-receptor-positive HER2-negative breast cancer; who were newly prescribed EVE 10 mg and EXE 25 mg; had Eastern Cooperative Oncology Group (ECOG) performance status of 0–1; and showed adequate renal function (serum creatinine level ≤ 1.5 × upper limit of normal). Patients with an edentulous jaw; oral mucositis within 1 mouth; chemotherapy administered within 1 month prior to randomization (except bisphosphonates or denosumab). The institutional review boards at each of the 31 study sites approved the study protocol. All patients provided written informed consent before the commencement of the study.

A total of 174 patients were randomly allocated to the two groups at enrolment and the treatment protocol (EVE 10 mg once a day and EXE 25 mg once a day) was initiated within 3 weeks from the date of enrolment to 169 patients, which consisted of the analysis population of the primary endpoint. “Protocol treatment completion” was defined as oral management for a period of 8 weeks in the control (C) and POC groups. The study protocol was registered online at the University Hospital Medical Information Network (UMIN), Japan (protocol ID 000016109) on January 5, 2015 and at ClinicalTrials.gov (NCT02376985).

#### Potential prognostic factors

The following categorical variables that could affect outcomes were used as covariates of analysis: (1) POC or C group; (2) Age <  65 or ≥ 65 years; (3) Use of BMA or not; (4) Chemotherapy or not; (5) BMI ≥ 25 kg/m^2^ or < 25 kg/m^2^; (6) Progesterone receptor (PgR)-positive or not; (7) visceral disease or not; (8) Metastatic or de novo; (8) Endocrine therapy 2 or 0–1; and (9) Incidence of oral mucositis within 8 weeks or no incidence.

#### Endpoints

In the original clinical trial, the primary endpoint was the comparison of incidence of grade 1 or worse oral mucositis between the POC and C groups evaluated by the oncologist over 8 weeks. The secondary endpoints were progression-free survival (PFS) and overall survival (OS) in all patients. PFS was defined as the interval between the first progression and the first day of EVE administration and OS was defined as the period of survival after the initiation of EVE treatment.

### Statistical analysis

Patient characteristics were summarized by mean and standard deviation (SD) for continuous factors and by count and proportion for categorical factors. The imbalance between treatment groups was tested by *t*-test or chi-square test. PFS and OS were estimated using the Kaplan-Meier method. Univariate and multivariate analyses for PFS were performed using the Cox proportional hazards model, in which 4 patients with unknown PgR status and 4 patients without BMI values were excluded (161 patients were included in total). Although, the primary endpoint of this trial was incidence of oral mucositis, sample size was not determined for PFS; however, at a total of 160 patients with the assumption that 40% of patients would be censored before observing progression, this trial had > 78% and > 64% power for detecting the hazard ratio of 2.0 and 1.5, respectively. All statistical analyses were performed using SAS version 9.4 (SAS Institute Inc., Cary, NC, USA) and significance was set at *P* < 0.05. All analyses were based on the data collected at a follow-up period of 13.9 months (median).

## Results

Patient characteristics are shown in Table 1. Baseline characteristics between POC and C groups were balanced. Univariate analysis for PFS showed that BMI ≥ 25 mg/m^2^ was associated with a significantly shorter PFS (HR: 1.85; 95% CI, 1.16–2.96, *P* = 0.010) and the presence of visceral disease (HR: 1.98; 95% CI 1.27–3.08, *P* = 0.002) was associated with a significantly shorter PFS. Multivariate analysis showed BMI ≥ 25 mg/m^2^ (HR: 1.84; 95% CI: 1.11–3.04, *P* = 0.018), the presence of visceral disease (HR: 2.13; 95% CI: 1.31–3.49, *P* = 0.003; Table 2), and BMA use were associated with a shorter PFS (HR: 1.71; 95% CI: 1.06–2.77, *P* = 0.028; Table 2).

**Table 1:** Patient characteristics

	POC Group (*n* = 82)			C Group (*n* = 87)			*P*-value
	n	%	95%CI	n	%	95%CI	
Age (years)							0.93
< 65	42	51.2	(39.9, 62.4)	44	50.6	(39.6, 61.5)	
≥ 65	40	48.8	(37.6, 60.1)	43	49.4	(38.5, 60.4)	
BMA							0.84
Not used	39	47.6	(36.4, 58.9)	40	46.0	(35.2, 57.0)	
Used	43	52.4	(41.1, 63.6)	47	54.0	(43.0, 64.8)	
Chemotherapy							0.55
Not used	74	90.2	(81.7, 95.7)	76	87.4	(78.5, 93.5)	
Used	8	9.8	(4.3, 18.3)	11	12.6	(6.5, 21.5)	
BMI (mg/m^2)^							0.07
< 25	54	65.9	(54.6, 76.0)	66	75.9	(65.5, 84.4)	
≥ 25	24	29.3	(19.7, 40.4)	21	24.1	(15.6, 34.5)	
Missing	4	4.9	(1.3, 12.0)	0	0.0	(0.0, 4.2)	
PgR							0.35
-	12	14.6	(7.8, 24.2)	13	14.9	(8.2, 24.2)	
+	65	79.3	(68.9, 87.4)	72	82.8	(73.2, 90.0)	
Unknown	2	2.4	(0.3, 8.5)	2	2.3	(0.3, 8.1)	
Missing	3	3.7	(0.3, 10.3)	0	0.0	(0. 4.2)	
Metastatic site							0.07
Non-visceral	34	41.5	(30.7, 52.9)	28	32.2	(22.6, 43.1)	
Visceral	45	54.9	(43.5, 65.9)	59	67.8	(56.9, 77.4)	
Missing	3	3.7	(0.8, 10.3)	0	0.0	(0.0, 4.2)	
Metastatic type							0.07
De novo	58	70.7	(59.6, 80.3)	72	82.8	(73.2, 90.0)	
Metastatic	21	25.6	(16.6, 36.4)	15	17.2	(10.0, 26.8)	
Missing	3	3.7	(0.8, 10.3)	0	0.0	(0.0, 4.2)	
Endocrine therapy for MBC							0.40
0	13	15.9	(8.7, 25.6)	8	9.2	(4.1, 17.3)	
1	17	20.7	(12.6, 31.1)	25	28.7	(19.5, 39.4)	
2	23	28.0	(18.7, 39.1)	27	31.0	(21.5, 41.9)	
3	29	35.4	(25.1, 46.7)	27	31.0	(21.5, 41.9)	
Incidence of oral mucositis within 8 weeks							0.02
No	20	24.4	(15.6, 35.1)	9	10.3	(4.8, 18.7)	
Yes	62	75.6	(64.9, 84.4)	78	89.7	(81.3, 95.2)

**Table 2:** Univariate and multivariate Cox proportional hazards regression models for progression free survival

	Univariate		Multivariate	
	HR (95% CI)	*P*-value	HR (95% CI)	*P*-value
D Group, B Group	0.97 (0.64, 1.46)	0.879	1.11 (0.72, 1.72)	0.628
Age (≥ 65, < 65 years)	0.97 (0.64, 1.46)	0.874	1.04 (0.66, 1.66)	0.862
Use of BMA, No use	1.26 (0.84, 1.89)	0.255	1.71 (1.06, 2.77)	0.028
Chemotherapy, No use	1.03 (0.55, 1.93)	0.930	1.19 (0.60, 2.37)	0.618
BMI ≥ 25, < 25	1.85 (1.16, 2.96)	0.010	1.84 (1.11, 3.04)	0.018
PgR+, −	1.02 (0.60, 1.72)	0.952	0.70 (0.38, 1.29)	0.257
Visceral, No visceral	1.98 (1.27, 3.08)	0.002	2.13 (1.31, 3.49)	0.003
Metastatic, De novo	1.05 (0.65, 1.69)	0.855	0.87 (0.49, 1.54)	0.625
Endocrine therapy 2, 0–1	1.34 (0.80, 2.22)	0.265	1.13 (0.60, 2.13)	0.701
Endocrine therapy 3, 0–1	1.22 (0.76, 1.96)	0.409	0.95 (0.56, 1.62)	0.856
Incidence of oral mucositis within 8 weeks, None	1.19 (0.69, 2.04)	0.536	1.41 (0.78, 2.57)	0.260

The median PFS was 5.57 months (95% CI, 4.62–6.72). The OS did not reach 50% over a median observational period of 13.9 months. The median PFS in the POC and C groups was 5.51 months (95% CI, 4.23–8.07) and 5.57 months (95% CI, 4.03–6.52), respectively.

**Fig. 1:**
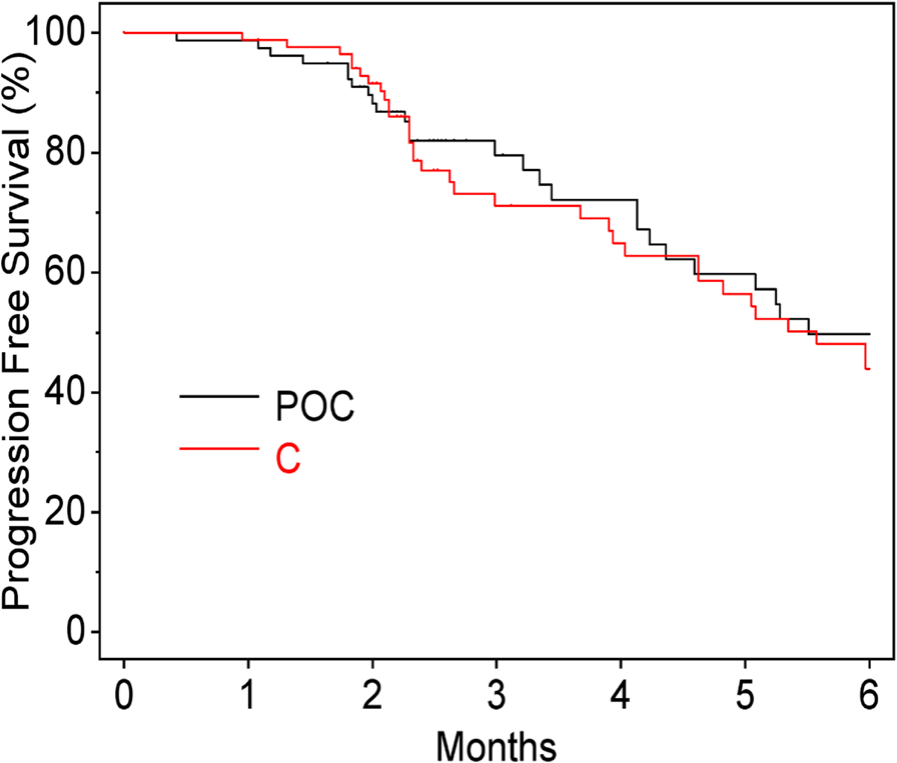
Progression-free survival. POC, professional oral care; C, control

**Fig. 2:**
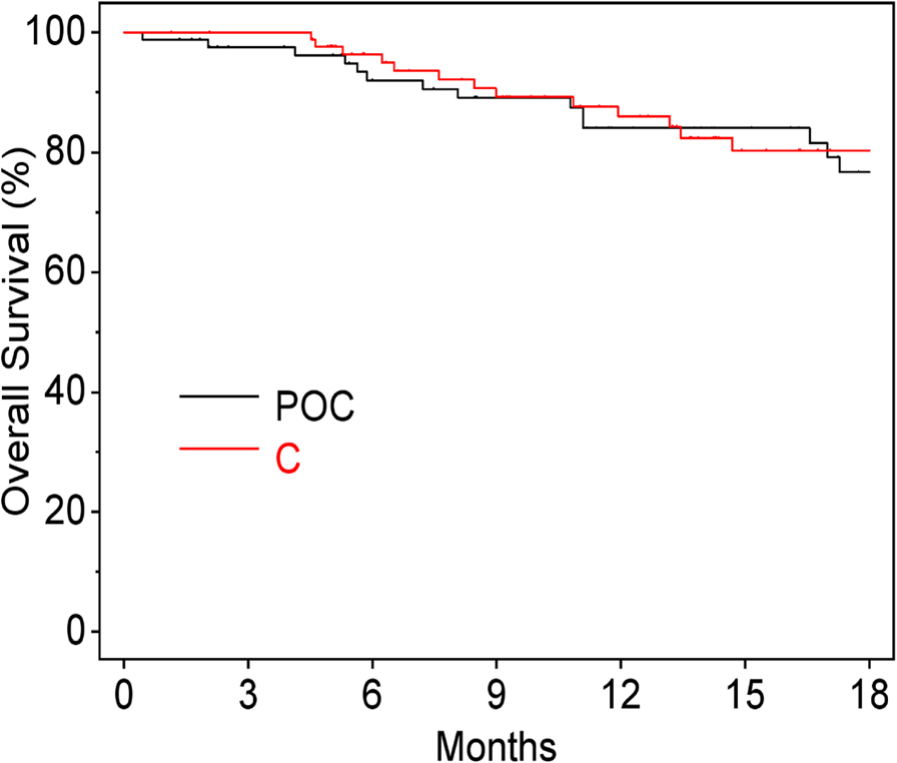
Overall survival. POC, professional oral care; C, control

**Fig. 3:**
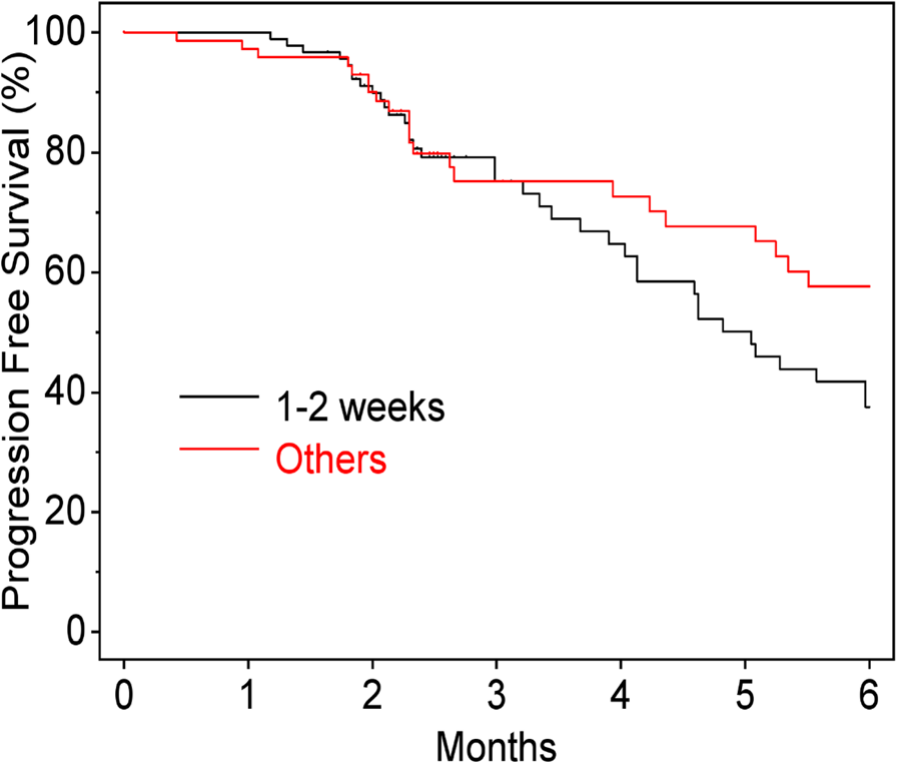
Progression-free survival (incidence of oral mucositis within two weeks)

There were no significant differences in PFS between the POC and C Groups (*P* = 0.801, HR 0.95, 95% CI, 0.63–1.42) (Fig. 1), or in OS between the groups (*P* = 0.971, HR: 0.99, 95% CI, 0.51–1.92) (Fig. 2). Additionally, there were no significant differences between the PFS of patients with oral mucositis within 2 weeks or not (*P* = 0.170, HR: 1.33, 95% CI, 0.89–1.98) (Fig. 3).

## Discussion

Novel therapies based on cyclin-dependent kinase (CDK)4/6 and phosphatidylinositol 3-kinase (PI3K) / protein kinase B (AKT) / mammalian target of rapamycin (mTOR) inhibitors have significantly improved prognosis for patients, increasing progression-free survival rate, and, in some cases, overall survival.

Our results showed a PFS of 5.6 months in patients receiving the combination treatment of EVE and EXE, which is shorter than the PFS reported in the BOLERO-2 trial (7.8 months), primarily since the Oral Care-BC trial had many patients with visceral disease.

A meta-analysis of stomatitis in patients receiving EVE showed that stomatitis within 8 weeks was associated with longer PFS in several trials [16]. However, we demonstrated that mucositis within 2 and 8 weeks was not associated with longer PFS. The reason for the differences in results remains unknown, however, it could be due to differences in primary cancer sites or the interaction with combination therapies. The meta-analysis included patients with advanced carcinoid tumors, pancreatic neuroendocrine tumors, renal cell carcinomas, and tuberous sclerosis complex, but our trial included only breast cancer patients. Similarly, the meta-analysis included EXE, vinorelbine, trastuzumab, and long-acting repeatable octreotide as combination drugs for EVE, but our trial included only EXE. Furthermore, patients in our trial were obliged to receive severe prophylactic oral care, but those in the meta-analysis did not always receive oral care.

We observed that non-visceral metastasis was associated with a longer PFS than visceral metastasis. However, Jose et al. reported that the effects of EVE and EXE did not differ between visceral and non-visceral metastasis in the BOLERO-2 trial [3]. This could be attributed to the differences in clinical trial design.

It has been reported that women with a high BMI receiving aromatase inhibitors in an adjuvant setting experience more recurrences than women with a low BMI [17–23]. We also showed that low BMI patients were associated with longer PFS than those with a high BMI, although there were differences in EVE exposure and trial design (adjuvant vs. metastatic setting). Obesity is most strongly associated with postmenopausal hormone receptor-positive breast cancer risk. Hormone replacement therapy (HRT) has been commonly used in Japan. A previous meta-analysis found that HRT recipients were at an increased risk of breast cancer, and since obesity is a side effect of HRT, it might influence our results.

Further, we discovered that non-bone-targeted therapy was a better prognostic factor of EVE and EXE. A recent meta-analysis reported that bone metastases (BMs) occur in 58% of patients with metastatic breast cancer [24]. BMs often cause severe bone pain and lead to bone fracture, known as skeletal-related events (SREs), including radiation or surgery to bone, fragile bone fracture, spinal cord compression, and hypercalcemia of bone metastasis [24]. SREs cause severe pain, impair mobility, reduce the quality of life (QoL), and increase mortality [25–28]. Patients receiving BMA had a poorer prognosis, including significant BM, prior to participating in our clinical trial.

The Safari study (UMIN000015168), a retrospective, multicenter cohort study, conducted in 1072 patients in Japan taking fulvestrant (500 mg) for ER-positive metastatic breast cancer showed that early line fulvestrant (500 mg) (F500) administration was associated with significantly longer time to treatment failure (TTF) than late line use [29, 30], however, EVE and EXE did not show this trend in our trial. In other words, EVE and EXE may be a promising drug combination regardless of treatment line.

Although the results of this clinical study are important, there were several limitations. First, the primary endpoint of this trial was the comparison of incidence of grade 1 or worse oral mucositis over 8 weeks between the POC and C groups, and not PFS and OS. Secondly, a larger sample size could have provided more reliable results. However, the trial was well-powered for detecting a sufficiently strong association with PFS, e.g. a hazard ratio of > 2.0, although in a post-hoc calculation, showed that BMI, use of BMA, and visceral involvement were important prognostic factors for progression and that the outcomes of the present trial are reliable. Lastly, a centralized data review of images and pathological examinations were not performed, as we felt that these were beyond the scope of this investigation. In future studies, a more extensive review of the literature could provide additional data to support our results.

## Conclusions

Professional oral care does not influence the prognosis of estrogen receptor-positive metastatic breast cancer patients. Non-visceral metastasis, BMI < 25 mg/m^2^, and those not receiving BMA might be good prognostic factors for patients receiving EVE and EXE.

## Data Availability

All data has been included in the manuscript.

## Abbreviations

POC: Professional oral care groups
C: Control groups
BMA: Bone modified agents
BMI: Body mass index
PgR: Progesterone receptor
MBC: Metastatic breast cancer

## References

[1] Hortobagyi GN, Chen D, Piccart M, Rugo HS, Burris HA, Pritchard KI (2016). Correlative analysis of genetic alterations and everolimus benefit in hormone receptor-positive, human epidermal growth factor receptor 2-negative advanced breast cancer: results from BOLERO-2. J Clin Oncol.

[2] Motzer RJ, Escudier B, Oudard S, Hutson TE, Porta C, Bracarda S (2008). Efficacy of everolimus in advanced renal cell carcinoma: a double-blind, randomised, placebo-controlled phase III trial. Lancet.

[3] Baselga J, Campone M, Piccart M, Burris HA, Rugo HS, Sahmoud T (2012). Everolimus in postmenopausal hormone-receptor-positive advanced breast cancer. N Engl J Med.

[4] Kandoth C, McLellan MD, Vandin F, Ye K, Niu B, Lu C (2013). Mutational landscape and significance across 12 major cancer types. Nature.

[5] Tesch H, Stoetzer OJ, Decker T, Murbacher CM, Neumeister R, Marme F (2015). 4EVER - Final efficacy analysis of the phase IIIb, multi-center, open label study for postmenopausal women with estrogen receptor positive locally advanced or metastatic breast cancer (BC) treated with everolimus (EVE) in combination with exemestane (EXE). Cancer Res.

[6] Fasching PA, Decker T, Schneeweiss A, Uleer C, Forster F, Wimberger P (2014). Breast cancer treatment with everolimus and exemestane for ER+ women - results of the 2nd interim analysis of the non-interventional trial BRAWO. Ann Oncol.

[7] Steger G, Bartsch R, Pfeiler G, Petru E, Greil R, Helfgott R (2017). Efficacy and safety of everolimus plus exemestane in HR+, HER2– advanced breast cancer progressing on/after prior endocrine therapy, in routine clinical practice: second interim analysis from STEPAUT. Cancer Res.

[8] Jerusalem G, de Boer RH, Hurvitz S, Yardley DA, Kovalenko E, Ejlertsen B (2018). Everolimus plus exemestane vs everolimus or capecitabine monotherapy for estrogen receptor-positive, HER2-negative advanced breast cancer: the BOLERO-6 randomized clinical trial. JAMA Oncol.

[9] Niikura N, Nakatukasa K, Amemiya T, Watanabe K-I, Hata H, Kikawa Y, et al. Oral Care Evaluation to Prevent oral mucositis in estrogen receptor-positive metastatic breast cancer patients treated with everolimus (Oral Care-BC): a randomized controlled phase III trial. Oncologist. 2020;2:223–30. 10.1634/theoncologist.2019-0382 PMC7011665 32043762

[10] Mukohara T (2015). PI3K mutations in breast cancer: prognostic and therapeutic implications. Breast Cancer.

[11] Cancer Genome Atlas Network (2012). Comprehensive molecular portraits of human breast tumours. Nature.

[12] Moynahan ME, Chen D, He W, Sung P, Samoila A, You D (2017). Correlation between PIK3CA mutations in cell-free DNA and everolimus efficacy in HR+, HER2- advanced breast cancer: results from BOLERO-2. Br J Cancer.

[13] Chandarlapaty S, Chen D, He W, Sung P, Samoila A, You D (2016). Prevalence of ESR1 mutations in cell-free DNA and outcomes in metastatic breast cancer: a secondary analysis of the BOLERO-2 clinical trial. JAMA Oncol.

[14] Sonis ST, Oster G, Fuchs H, Bellm L, Bradford WZ, Edelsberg J (2001). Oral mucositis and the clinical and economic outcomes of hematopoietic stem-cell transplantation. J Clin Oncol.

[15] Elting LS, Cooksley C, Chambers M, Cantor SB, Manzullo E, Rubenstein EB (2003). The burdens of cancer therapy. Clinical and economic outcomes of chemotherapy-induced mucositis. Cancer.

[16] Rugo HS, Hortobagyi GN, Yao J, Pavel M, Ravaud A, Franz D (2016). Meta-analysis of stomatitis in clinical studies of everolimus: incidence and relationship with efficacy. Ann Oncol.

[17] Loi S, Milne RL, Friedlander ML, McCredie MR, Giles GG, Hopper JL (2005). Obesity and outcomes in premenopausal and postmenopausal breast cancer. Cancer Epidemiol Biomarkers Prev.

[18] Ellsworth RE, Ellsworth CD, Shriver DL, Henry CD, Jackson M (2011). Effect of obesity on gene expression in invasive breast tumors. Cancer Res.

[19] Jiralerspong S, Wieand T, Rimawi MF (2011). Obesity, adjuvant therapy, and survival outcomes in early-stage breast cancer. Cancer Res.

[20] Kwan ML, Chen WY, Kroenke CH, Weltzien E, Beasley JM, Nechuta SJ (2011). Pre-diagnosis body mass index and breast cancer prognosis and survival: report from the after Breast Cancer Pooling Project. Cancer Res.

[21] Sestak I, Distler W, Forbes JF, Dowsett M, Howell A, Cuzick J (2010). Effect of body mass index on recurrences in tamoxifen and anastrozole treated women: an exploratory analysis from the ATAC trial. J Clin Oncol.

[22] Pfeiler G, Königsberg R, Fesl C, Mlineritsch B, Stoeger H, Singer CF (2011). Impact of body mass index on the efficacy of endocrine therapy in premenopausal patients with breast cancer: an analysis of the prospective ABCSG-12 trial. J Clin Oncol.

[23] Sendur MAN, Aksoy S, Zengin N, Altundag K (2012). Efficacy of adjuvant aromatase inhibitor in hormone receptor-positive postmenopausal breast cancer patients according to the body mass index. Br J Cancer.

[24] Body JJ, Quinn G, Talbot S, Booth E, Demonty G, Taylor A (2017). Systematic review and meta-analysis on the proportion of patients with breast cancer who develop bone metastases. Crit Rev Oncol Hematol.

[25] Coleman RE (2006). Clinical features of metastatic bone disease and risk of skeletal morbidity. Clin Cancer Res.

[26] Coleman RE (2001). Metastatic bone disease: clinical features, pathophysiology and treatment strategies. Cancer Treat Rev.

[27] Costa L, Badia X, Chow E, Lipton A, Wardley A (2008). Impact of skeletal complications on patients quality of life, mobility, and functional independence. Supp Care Cancer.

[28] von Moos R, Body JJ, Egerdi B, Stopeck A, Brown J, Fallowfield L (2016). Pain and analgesic use associated with skeletal-related events in patients with advanced cancer and bone metastases. Supp Care Cancer.

[29] Kawaguchi H, Masuda N, Nakayama T, Aogi K, Anan K, Ito Y (2017). Outcomes of fulvestrant therapy among japanese women with advanced breast cancer: a retrospective multicenter cohort study (JBCRG-C06; Safari). Breast Cancer Res Treat.

[30] Kawaguchi H, Masuda N, Nakayama T, Aogi K, Anan K, Ito Y (2018). Factors associated with prolonged time to treatment failure with fulvestrant 500 mg in patients with post-menopausal estrogen receptor-positive advanced breastcancer: a sub-group analysis of the JBCRG-C06 Safari study. Curr Med Res Opin.

